# Ethyl 2-(3,5-dimethyl-1,1-dioxo-2*H*-1λ^6^,2,6-thia­diazin-4-yl)benzoate

**DOI:** 10.1107/S1600536812024907

**Published:** 2012-06-20

**Authors:** Nilay Bhatt, Pralav Bhatt, Kartik B. Vyas, Kiran Nimavat, Thavendran Govender, Hendrik G. Kruger, Glenn E. M. Maguire

**Affiliations:** a Chemistry Department, JJT University, Rajasthan, India; b School of Chemistry, University of KwaZulu-Natal, Durban 4000, South Africa; c Sheth L.H. Science College, Mansa, Gujarat, India; d Department of Chemistry, Government Science College, Gandhinagar, Gujarat, India; e School of Pharmacology, University of KwaZulu-Natal, Westville Campus, Private Bag-X54001, Durban, South Africa

## Abstract

In the title compound, C_14_H_16_N_2_O_4_S, the thia­diazine ring is in a half-boat conformation. The aromatic ring deviates from the plane of this moiety at an angle of 74.6 (2)°. The structure displays inter­molecular N—H⋯O hydrogen bonding [N⋯O = 2.8157 (16) Å], creating ribbons along the [010] axis. There are also weak C—H⋯O inter­actions in the crystal but no π–π stacking.

## Related literature

For the synthesis of 1,2,6-thia­diazine-1,1-dioxide derivatives, see: Wright (1964 ▶); Ochoa & Stud (1978 ▶). For the biological activity of 1,2,6-thia­diazine-1,1-dioxide derivatives, see: Aran *et al.* (1986 ▶); Herrero *et al.* (1992 ▶); Breining *et al.* (1995 ▶); Campillo *et al.* (2000 ▶). For related structures, see: Elguero *et al.* (1982 ▶). 
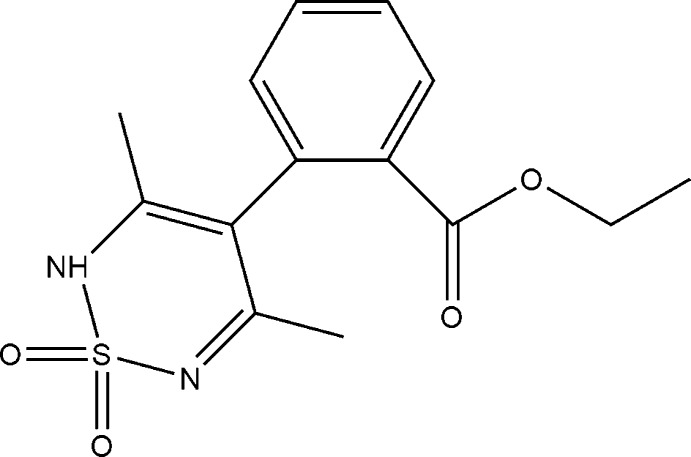


## Experimental

### 

#### Crystal data

C_14_H_16_N_2_O_4_S
*M* *_r_* = 308.35
Monoclinic, 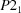

*a* = 10.3943 (2) Å
*b* = 6.6089 (2) Å
*c* = 10.6563 (3) Å
β = 94.982 (2)°
*V* = 729.27 (3) Å^3^
*Z* = 2
Mo *K*α radiation
μ = 0.24 mm^−1^
*T* = 173 K
0.25 × 0.24 × 0.23 mm

#### Data collection

Nonius KappaCCD diffractometer
Absorption correction: multi-scan (*SADABS*; Bruker, 2006 ▶) *T*_min_ = 0.943, *T*_max_ = 0.947
3321 measured reflections
3321 independent reflections
3083 reflections with *I* > 2σ(*I*)
*R*_int_ = 0.013

#### Refinement

*R*[*F* ^2^ > 2σ(*F* ^2^)] = 0.027
*wR*(*F* ^2^) = 0.067
*S* = 1.08
3321 reflections
198 parameters
2 restraints
H atoms treated by a mixture of independent and constrained refinement
Δρ_max_ = 0.22 e Å^−3^
Δρ_min_ = −0.22 e Å^−3^
Absolute structure: Flack (1983 ▶) 1512 Friedel pairs
Flack parameter: −0.03 (5)

### 

Data collection: *COLLECT* (Nonius, 2000 ▶); cell refinement: *DENZO-SMN* (Otwinowski & Minor, 1997 ▶); data reduction: *DENZO-SMN*; program(s) used to solve structure: *SHELXS97* (Sheldrick, 2008 ▶); program(s) used to refine structure: *SHELXL97* (Sheldrick, 2008 ▶); molecular graphics: *OLEX2* (Dolomanov *et al.*, 2009 ▶); software used to prepare material for publication: *SHELXL97*.

## Supplementary Material

Crystal structure: contains datablock(s) I, global. DOI: 10.1107/S1600536812024907/hg5223sup1.cif

Structure factors: contains datablock(s) I. DOI: 10.1107/S1600536812024907/hg5223Isup2.hkl

Supplementary material file. DOI: 10.1107/S1600536812024907/hg5223Isup3.cml

Additional supplementary materials:  crystallographic information; 3D view; checkCIF report

## Figures and Tables

**Table 1:** Hydrogen-bond geometry (Å, °)

*D*—H⋯*A*	*D*—H	H⋯*A*	*D*⋯*A*	*D*—H⋯*A*
N1—H1⋯O3^i^	0.97 (2)	1.85 (2)	2.8157 (16)	175 (2)
C5—H5*A*⋯O1^ii^	0.98	2.52	3.310 (2)	137

Symmetry codes: (i) 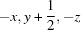; (ii) 
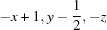
.

**Fig. 1.**
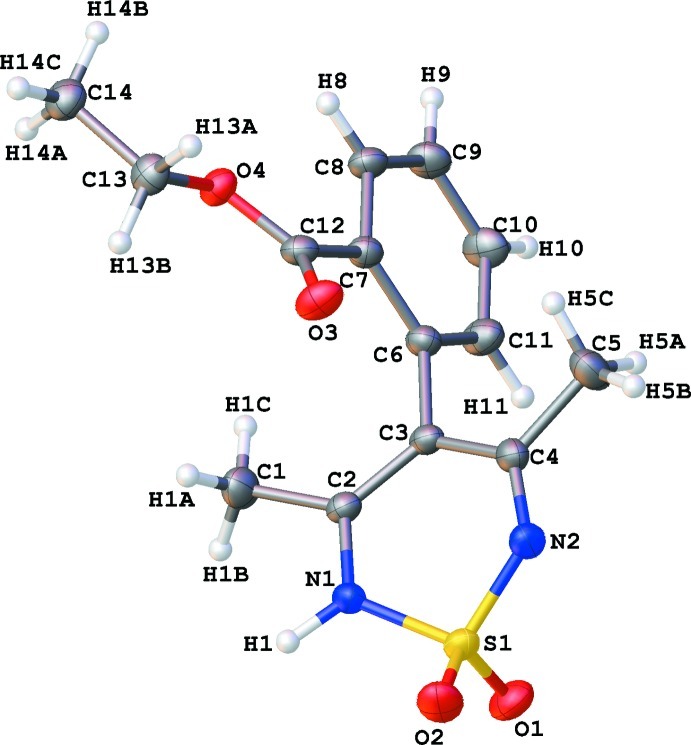
The molecular structure of the title compound with atomic numbering scheme. The H atoms have been omitted for clarity. Displacement ellipsoids are drawn at 40% probability.

**Fig. 2.**
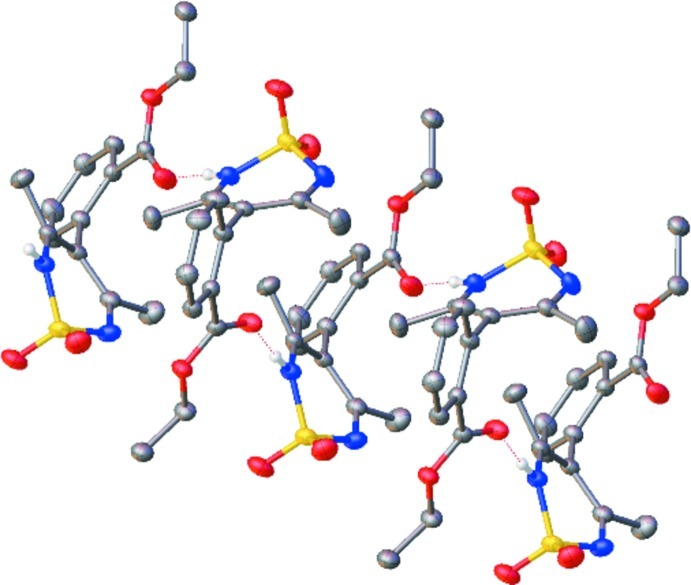
The hydrogen bonding ineractions of the title compound along the [111] axis. All H atoms except those involved in hydrogen bonding interactions have been omitted for clarity.

## supplementary crystallographic information

### Comment

The synthesis of 1,2,6-thiadiazine-1,1-dioxides derivatives was first reported using sulfamide with alpha and beta diketones (Wright, 1964). Purine and pyrimidine nucleotide versions of this structure have also been synthesized (Ochoa & Stud, 1978). More recently 1,2,6-thiadiazine-1,1-dioxide derivatives have since been reported to posses antiparasitic (Aran *et al.*, 1986), antiprotozoal (Herrero *et al.*, 1992), anti-HIV-1 activity (Breining *et al.*, 1995) and also act as bronchodilators (Campillo *et al.*, 2000).

The first single-crystal X-ray structure of a 3,5 dimethyl-1,2,6-thiadiazine-1,1-dioxide derivative was reported by Elguero *et al.*, 1982. The structure displayed intermolecular hydrogen bonding at N(2)—H(1)—O(2), 2.904 Å. The title compound is the first 3,5 dimethyl based structure reported with an aromatic ring at position 4 of the thiadiazine ring. It is also the first containing an ester functional group in the broader family of 1,2,6-thiadiazine-1,1-dioxides. The sulfur atom deviates from the plane of the ring by 0.53 Å (Fig. 1). The aromatic ring is nearly orthogonal to the thiadiazine ring with an angle of 74.6 (2)° from the plane. The structure displays intermolecular hydrogen bonding N(1)—H(1)—O(3), 2.8157 (16)Å creating ribbons along the [010] axis (Fig. 2). There is no π -π stacking in the crystal structure.

### Experimental

To ethanol (25 ml) and 2-(3,5-dimethyl-1,1-dioxo-2*H*-1,2,6-thiadiazin-4-yl) benzoic acid (10 m*M*) was slowly added thionyl chloride (50 m*M*), the contents were refluxed for 4 h, untill the reaction was complete (TLC *R*_f_ = 0.7 in 80% ethyl acetate/hexane). The contents were filtered. The filtrate was evaporated under reduced pressure yielding a clear oil. To this residue was added a solution of ethanol/ethyl acetate, (10 ml) (10/90) to yield a white colourless solid (55%). *M*.p.= 417.5 K.

Crystals suitable for X-ray analysis were grown in methanol/ethyl acetate at room temprature.

### Refinement

All hydrogen atoms were positioned geometrically with d(C-H) ranging from 0.95 Å to 0.99 Å and d(N-H) = 0.88 Å and refined as riding on their parent atoms with U_iso_ (H) = 1.2 - 1.5 U_eq_ (C ). The hydrogen atom H1 was located in the difference electron density maps and refined with O—H distance restraint to the value of 0.97 (1)Å.

### Crystal data

**Table:**

C_14_H_16_N_2_O_4_S	*F*(000) = 324
*M**_r_* = 308.35	*D*_x_ = 1.404 Mg m^−^^3^
Monoclinic, *P*2_1_	Mo *K*α radiation, λ = 0.71073 Å
Hall symbol: P 2yb	Cell parameters from 3321 reflections
*a* = 10.3943 (2) Å	θ = 3.6–27.5°
*b* = 6.6089 (2) Å	µ = 0.24 mm^−^^1^
*c* = 10.6563 (3) Å	*T* = 173 K
β = 94.982 (2)°	Block, colourless
*V* = 729.27 (3) Å^3^	0.25 × 0.24 × 0.23 mm
*Z* = 2	

### Data collection

**Table:**

Nonius KappaCCD diffractometer	3321 independent reflections
Radiation source: fine-focus sealed tube	3083 reflections with *I* > 2σ(*I*)
Graphite monochromator	*R*_int_ = 0.013
1.2° φ scans and ω scans	θ_max_ = 27.5°, θ_min_ = 3.6°
Absorption correction: multi-scan (*SADABS*; Bruker, 2006)	*h* = −13→13
*T*_min_ = 0.943, *T*_max_ = 0.947	*k* = −8→8
3321 measured reflections	*l* = −13→13

### Refinement

**Table:**

Refinement on *F*^2^	Hydrogen site location: inferred from neighbouring sites
Least-squares matrix: full	H atoms treated by a mixture of independent and constrained refinement
*R*[*F*^2^ > 2σ(*F*^2^)] = 0.027	*w* = 1/[σ^2^(*F*_o_^2^) + (0.0356*P*)^2^ + 0.1059*P*] where *P* = (*F*_o_^2^ + 2*F*_c_^2^)/3
*wR*(*F*^2^) = 0.067	(Δ/σ)_max_ < 0.001
*S* = 1.08	Δρ_max_ = 0.22 e Å^−^^3^
3321 reflections	Δρ_min_ = −0.22 e Å^−^^3^
198 parameters	Extinction correction: *SHELXL97* (Sheldrick, 2008), Fc^*^=kFc[1+0.001xFc^2^λ^3^/sin(2θ)]^-1/4^
2 restraints	Extinction coefficient: 0.021 (3)
Primary atom site location: structure-invariant direct methods	Absolute structure: Flack (1983) ???? Friedel pairs
Secondary atom site location: difference Fourier map	Flack parameter: −0.03 (5)

### Special details

**Table:**

Geometry. All e.s.d.'s (except the e.s.d. in the dihedral angle between two l.s. planes) are estimated using the full covariance matrix. The cell e.s.d.'s are taken into account individually in the estimation of e.s.d.'s in distances, angles and torsion angles; correlations between e.s.d.'s in cell parameters are only used when they are defined by crystal symmetry. An approximate (isotropic) treatment of cell e.s.d.'s is used for estimating e.s.d.'s involving l.s. planes.
Refinement. Refinement of *F*^2^ against ALL reflections. The weighted *R*-factor *wR* and goodness of fit *S* are based on *F*^2^, conventional *R*-factors *R* are based on *F*, with *F* set to zero for negative *F*^2^. The threshold expression of *F*^2^ > σ(*F*^2^) is used only for calculating *R*-factors(gt) *etc*. and is not relevant to the choice of reflections for refinement. *R*-factors based on *F*^2^ are statistically about twice as large as those based on *F*, and *R*- factors based on ALL data will be even larger.

### Fractional atomic coordinates and isotropic or equivalent isotropic displacement parameters (Å^2^)

**Table:**

	*x*	*y*	*z*	*U*_iso_*/*U*_eq_	
S1	0.22417 (3)	0.66320 (6)	−0.19373 (3)	0.02436 (10)	
O1	0.32997 (11)	0.7730 (2)	−0.23716 (11)	0.0362 (3)	
O2	0.13244 (11)	0.58238 (18)	−0.28774 (10)	0.0343 (3)	
O3	0.07360 (10)	0.52605 (18)	0.21011 (10)	0.0305 (3)	
O4	0.04265 (9)	0.69099 (17)	0.38957 (9)	0.0245 (2)	
N1	0.14372 (11)	0.81211 (19)	−0.10443 (11)	0.0228 (3)	
H1	0.0717 (13)	0.887 (3)	−0.1454 (17)	0.049 (6)*	
N2	0.27760 (12)	0.4887 (2)	−0.09828 (12)	0.0287 (3)	
C1	0.11769 (16)	1.0140 (3)	0.07966 (15)	0.0323 (4)	
H1A	0.0268	0.9787	0.0850	0.048*	
H1B	0.1233	1.1368	0.0288	0.048*	
H1C	0.1591	1.0381	0.1645	0.048*	
C2	0.18463 (13)	0.8442 (2)	0.01961 (13)	0.0209 (3)	
C3	0.27298 (13)	0.7164 (2)	0.08085 (13)	0.0206 (3)	
C4	0.30819 (13)	0.5334 (2)	0.02143 (14)	0.0244 (3)	
C5	0.37968 (18)	0.3714 (3)	0.09778 (16)	0.0377 (4)	
H5A	0.4693	0.4140	0.1186	0.057*	
H5B	0.3784	0.2457	0.0489	0.057*	
H5C	0.3380	0.3487	0.1756	0.057*	
C6	0.32137 (14)	0.7514 (2)	0.21576 (13)	0.0226 (3)	
C7	0.24812 (13)	0.7118 (2)	0.31791 (12)	0.0207 (3)	
C8	0.30187 (14)	0.7466 (2)	0.44105 (13)	0.0243 (3)	
H8	0.2517	0.7216	0.5098	0.029*	
C9	0.42703 (15)	0.8167 (3)	0.46405 (14)	0.0284 (3)	
H9	0.4634	0.8364	0.5481	0.034*	
C10	0.49884 (15)	0.8579 (3)	0.36372 (15)	0.0313 (4)	
H10	0.5843	0.9086	0.3788	0.038*	
C11	0.44671 (14)	0.8255 (3)	0.24139 (14)	0.0296 (3)	
H11	0.4973	0.8543	0.1734	0.036*	
C12	0.11380 (13)	0.6337 (2)	0.29881 (12)	0.0222 (3)	
C13	−0.08984 (13)	0.6165 (2)	0.38507 (14)	0.0269 (3)	
H13A	−0.0908	0.4668	0.3881	0.032*	
H13B	−0.1394	0.6610	0.3064	0.032*	
C14	−0.14763 (15)	0.7028 (3)	0.49745 (14)	0.0309 (4)	
H14A	−0.1467	0.8509	0.4928	0.046*	
H14B	−0.0971	0.6586	0.5745	0.046*	
H14C	−0.2369	0.6554	0.4986	0.046*	

### Atomic displacement parameters (Å^2^)

**Table:**

	*U*^11^	*U*^22^	*U*^33^	*U*^12^	*U*^13^	*U*^23^
S1	0.02388 (17)	0.0332 (2)	0.01587 (16)	0.00301 (16)	0.00125 (11)	−0.00161 (15)
O1	0.0280 (6)	0.0546 (7)	0.0272 (6)	−0.0033 (5)	0.0091 (5)	0.0012 (5)
O2	0.0371 (6)	0.0418 (7)	0.0223 (5)	0.0019 (5)	−0.0074 (5)	−0.0080 (5)
O3	0.0283 (5)	0.0411 (7)	0.0221 (5)	−0.0120 (5)	0.0017 (4)	−0.0079 (5)
O4	0.0196 (4)	0.0317 (6)	0.0224 (5)	−0.0035 (4)	0.0033 (4)	−0.0038 (5)
N1	0.0220 (6)	0.0292 (7)	0.0169 (6)	0.0044 (5)	0.0001 (4)	0.0016 (5)
N2	0.0318 (7)	0.0337 (7)	0.0203 (6)	0.0090 (6)	0.0013 (5)	−0.0023 (5)
C1	0.0378 (9)	0.0303 (9)	0.0288 (8)	0.0088 (7)	0.0025 (7)	−0.0046 (7)
C2	0.0210 (7)	0.0248 (7)	0.0170 (7)	−0.0015 (6)	0.0030 (5)	−0.0003 (6)
C3	0.0184 (6)	0.0282 (8)	0.0153 (6)	−0.0006 (5)	0.0016 (5)	0.0006 (5)
C4	0.0204 (7)	0.0310 (8)	0.0217 (7)	0.0042 (6)	0.0025 (5)	0.0026 (6)
C5	0.0416 (10)	0.0385 (10)	0.0317 (9)	0.0143 (8)	−0.0046 (7)	0.0036 (7)
C6	0.0218 (7)	0.0278 (7)	0.0178 (7)	0.0009 (6)	−0.0002 (5)	0.0017 (6)
C7	0.0218 (6)	0.0225 (8)	0.0175 (6)	−0.0007 (5)	−0.0007 (5)	−0.0009 (5)
C8	0.0278 (7)	0.0284 (8)	0.0164 (7)	−0.0008 (6)	0.0009 (5)	−0.0006 (6)
C9	0.0286 (8)	0.0337 (8)	0.0216 (7)	−0.0037 (7)	−0.0051 (6)	−0.0019 (6)
C10	0.0230 (7)	0.0416 (10)	0.0282 (8)	−0.0078 (7)	−0.0042 (6)	0.0004 (7)
C11	0.0232 (7)	0.0420 (9)	0.0238 (8)	−0.0039 (7)	0.0027 (6)	0.0016 (7)
C12	0.0240 (7)	0.0255 (8)	0.0169 (6)	−0.0020 (6)	0.0010 (5)	0.0023 (6)
C13	0.0201 (7)	0.0315 (9)	0.0290 (8)	−0.0046 (6)	0.0016 (5)	−0.0024 (6)
C14	0.0263 (7)	0.0362 (10)	0.0310 (8)	−0.0025 (6)	0.0062 (6)	−0.0002 (7)

### Geometric parameters (Å, º)

**Table:**

S1—O2	1.4255 (11)	C5—H5B	0.9800
S1—O1	1.4279 (12)	C5—H5C	0.9800
S1—N2	1.6046 (14)	C6—C11	1.396 (2)
S1—N1	1.6466 (12)	C6—C7	1.406 (2)
O3—C12	1.2269 (17)	C7—C8	1.3998 (18)
O4—C12	1.3229 (17)	C7—C12	1.4859 (19)
O4—C13	1.4593 (16)	C8—C9	1.383 (2)
N1—C2	1.3694 (18)	C8—H8	0.9500
N1—H1	0.9697 (10)	C9—C10	1.383 (2)
N2—C4	1.3210 (19)	C9—H9	0.9500
C1—C2	1.494 (2)	C10—C11	1.384 (2)
C1—H1A	0.9800	C10—H10	0.9500
C1—H1B	0.9800	C11—H11	0.9500
C1—H1C	0.9800	C13—C14	1.498 (2)
C2—C3	1.3707 (19)	C13—H13A	0.9900
C3—C4	1.427 (2)	C13—H13B	0.9900
C3—C6	1.4992 (19)	C14—H14A	0.9800
C4—C5	1.502 (2)	C14—H14B	0.9800
C5—H5A	0.9800	C14—H14C	0.9800
			
O2—S1—O1	116.72 (7)	C11—C6—C3	118.12 (13)
O2—S1—N2	110.47 (7)	C7—C6—C3	123.68 (13)
O1—S1—N2	109.69 (7)	C8—C7—C6	119.76 (13)
O2—S1—N1	106.73 (7)	C8—C7—C12	118.63 (12)
O1—S1—N1	109.12 (7)	C6—C7—C12	121.60 (12)
N2—S1—N1	103.20 (6)	C9—C8—C7	120.93 (13)
C12—O4—C13	117.73 (11)	C9—C8—H8	119.5
C2—N1—S1	121.31 (10)	C7—C8—H8	119.5
C2—N1—H1	121.2 (12)	C8—C9—C10	119.44 (14)
S1—N1—H1	117.2 (12)	C8—C9—H9	120.3
C4—N2—S1	119.50 (11)	C10—C9—H9	120.3
C2—C1—H1A	109.5	C9—C10—C11	120.28 (14)
C2—C1—H1B	109.5	C9—C10—H10	119.9
H1A—C1—H1B	109.5	C11—C10—H10	119.9
C2—C1—H1C	109.5	C10—C11—C6	121.38 (14)
H1A—C1—H1C	109.5	C10—C11—H11	119.3
H1B—C1—H1C	109.5	C6—C11—H11	119.3
N1—C2—C3	120.21 (13)	O3—C12—O4	123.68 (12)
N1—C2—C1	114.38 (13)	O3—C12—C7	124.07 (13)
C3—C2—C1	125.23 (13)	O4—C12—C7	112.25 (11)
C2—C3—C4	119.86 (13)	O4—C13—C14	106.74 (11)
C2—C3—C6	121.10 (13)	O4—C13—H13A	110.4
C4—C3—C6	118.59 (13)	C14—C13—H13A	110.4
N2—C4—C3	124.64 (13)	O4—C13—H13B	110.4
N2—C4—C5	115.46 (14)	C14—C13—H13B	110.4
C3—C4—C5	119.84 (13)	H13A—C13—H13B	108.6
C4—C5—H5A	109.5	C13—C14—H14A	109.5
C4—C5—H5B	109.5	C13—C14—H14B	109.5
H5A—C5—H5B	109.5	H14A—C14—H14B	109.5
C4—C5—H5C	109.5	C13—C14—H14C	109.5
H5A—C5—H5C	109.5	H14A—C14—H14C	109.5
H5B—C5—H5C	109.5	H14B—C14—H14C	109.5
C11—C6—C7	118.20 (13)		
			
O2—S1—N1—C2	151.55 (12)	C2—C3—C6—C7	74.6 (2)
O1—S1—N1—C2	−81.50 (13)	C4—C3—C6—C7	−97.68 (17)
N2—S1—N1—C2	35.09 (13)	C11—C6—C7—C8	−0.2 (2)
O2—S1—N2—C4	−146.56 (12)	C3—C6—C7—C8	179.14 (14)
O1—S1—N2—C4	83.40 (13)	C11—C6—C7—C12	179.72 (14)
N1—S1—N2—C4	−32.79 (13)	C3—C6—C7—C12	−0.9 (2)
S1—N1—C2—C3	−16.62 (19)	C6—C7—C8—C9	−0.9 (2)
S1—N1—C2—C1	167.96 (11)	C12—C7—C8—C9	179.14 (14)
N1—C2—C3—C4	−9.2 (2)	C7—C8—C9—C10	1.7 (2)
C1—C2—C3—C4	165.70 (14)	C8—C9—C10—C11	−1.2 (3)
N1—C2—C3—C6	178.59 (13)	C9—C10—C11—C6	0.1 (3)
C1—C2—C3—C6	−6.5 (2)	C7—C6—C11—C10	0.6 (2)
S1—N2—C4—C3	13.8 (2)	C3—C6—C11—C10	−178.75 (15)
S1—N2—C4—C5	−169.16 (12)	C13—O4—C12—O3	1.8 (2)
C2—C3—C4—N2	11.2 (2)	C13—O4—C12—C7	−177.25 (12)
C6—C3—C4—N2	−176.42 (14)	C8—C7—C12—O3	−150.10 (15)
C2—C3—C4—C5	−165.74 (14)	C6—C7—C12—O3	30.0 (2)
C6—C3—C4—C5	6.7 (2)	C8—C7—C12—O4	28.97 (19)
C2—C3—C6—C11	−106.03 (17)	C6—C7—C12—O4	−150.95 (13)
C4—C3—C6—C11	81.65 (18)	C12—O4—C13—C14	179.87 (12)

### Hydrogen-bond geometry (Å, º)

**Table:**

*D*—H···*A*	*D*—H	H···*A*	*D*···*A*	*D*—H···*A*
N1—H1···O3^i^	0.97 (2)	1.85 (2)	2.8157 (16)	175 (2)
C5—H5*A*···O1^ii^	0.98	2.52	3.310 (2)	137

Symmetry codes: (i) −*x*, *y*+1/2, −*z*; (ii) −*x*+1, *y*−1/2, −*z*.
